# Utility of the prostest to predict and monitor response to chemotherapy in metastatic Castration-Resistant prostate cancer: A prospective pilot study

**DOI:** 10.1186/s12885-025-14549-3

**Published:** 2025-07-10

**Authors:** Kambiz Rahbar, Katrin Schlack, Martin Bögemann

**Affiliations:** 1 Department of Nuclear Medicine, University Hospital Muenster, Albert-Schweitzer-Campus 1, Muenster, 48149 Germany; 2 Department of Urology, University Hospital Muenster, Muenster, Germany; 3 West German Cancer Centre, Hufelandstrasse 55, Essen, 45147 Germany

**Keywords:** PROSTest, Prostate cancer, Docetaxel

## Abstract

**Background:**

Metastatic castration-resistant prostate cancer (mCRPC) treatment has advanced with therapies such as androgen receptor pathway inhibitors (ARPIs), taxane chemotherapies like docetaxel, and novel agents like ^177^Lu-PSMA. However, predicting patient responses and sequencing therapies remain critical unmet needs. This study investigated whether the PROSTest, a molecular assay analyzing prostate cancer-specific transcripts, could predict treatment response or prognosis in mCRPC patients receiving docetaxel, compared to changes in prostate-specific antigen (PSA) levels.

**Methods:**

Nineteen patients with mCRPC were treated with a median of 10 docetaxel cycles. PROSTest, which measures tumor-related signaling pathways, was analyzed via qPCR, while PSA levels were determined using standard clinical assays. PSA and PROSTest scores were measured at baseline and after one treatment cycle. PROSTest, which evaluates tumor-related signaling pathways, was analyzed via qPCR, and prostate-specific antigen (PSA) levels were measured using standard clinical assays. Responses were categorized per Prostate Cancer Working Group 3-criteria. Associations between biomarker changes, progression-free survival (PFS), and overall survival (OS) were evaluated using Kaplan-Meier survival analysis and hazard ratios (HRs).

**Results:**

Baseline PSA and PROSTest scores did not predict response or survival. However, early changes in both biomarkers after one treatment cycle significantly correlated with PSA-PFS. PROSTest changes demonstrated a stronger association with PFS (HR: 0.04) compared to PSA (HR: 0.2). Early changes in PROSTest were also significantly (*p* = 0.03, AUC = 0.81 ± 0.14) associated with OS in contrast to PSA changes.

**Conclusions:**

This pilot study suggests that PROSTest may offer superior predictive and prognostic value compared to PSA in mCRPC patients undergoing docetaxel therapy. Larger, multicenter studies are needed to confirm these findings and validate the assay’s clinical integration.

**Trial registration:**

NCT06872619.

## Introduction

Significant advances have been made in the treatment of metastatic castration-resistant prostate cancer (mCRPC) with the development of several new agents (e.g., abiraterone and enzalutamine (and combinations) as well as ^177^Lu-PSMA [1, 2]. Optimal sequencing of the different therapeutic strategies remains a key unmet need [3]. Currently, therapy choice is based on a patient’s clinical and genetic profile (tumor molecular profile), the availability of molecular imaging, and the safety profiles of the agents. While androgen receptor pathway inhibitors (ARPIs) as well as the taxane chemotherapy docetaxel are mainstays of first-line treatment for mCRPC, ARPIs are favored over docetaxel for first-line treatment based on safety and side-effect profile with docetaxel typically relegated to the second line. More recently, triplet (ARPI + docetaxel + Androgen deprivation) may provide more benefit than single or double therapeutic approaches [4]. Docetaxel, a microtubule-stabilizing taxane chemotherapy, was the first drug to show survival benefit in the treatment of mCRPC [5]. Nevertheless, identifying who will or may not respond is an important aspect of therapy choice.

Changes in PSA are typically used to help monitor treatment responses after a therapy has been initiated. Guidelines have emphasized measurement of PSA decline [1, 6] as one method for evaluating responses, but there are significant limitations, including the phenomenon of an initial PSA surge under therapy [7, 8]; the latter occurs in approximately 5–20% of responders. Studies indicate that the PSA-response rate (defined as a decrease of *≥* 50%) to docetaxal may occur in 25–50% of patients [9–11]. Determining whether to continue this therapy or when to transition to other agents (e.g., cabazitaxel and abiraterone) remains a challenge [12].

The recently developed test, PROSTest [13, 14], utilizes 30 prostate cancer (PCa) transcripts detected by qPCR in peripheral whole blood samples. This assay produces an output scaled from 0 to 100, where algorithmic scores of *≥* 50 are indicative of PCa with high sensitivity; it appears to be more accurate than PSA for tumor detection. The assay was developed to capture PCa-related signaling pathways associated with tumor pathobiology, such as proliferation and metabolism. These biological signals differ fundamentally from PSA, which is a secretory biomarker and a surrogate for tumor mass. A key issue with this marker is that not all tumors secrete it, particularly in later stages tumors, and baseline levels can be low (< 2ng/mL), making it difficult to monitor significant decreases.

We hypothesized that changes in transcript levels after initiation of docetaxel chemotherapy in a cohort of patients with mCRPC may have utility for predicting response or early treatment failure to this agent. We also examined whether this molecular tool had prognostic value by examining the relationship between early biomarker changes and overall survival (OS). We directly compared how the PROSTest functioned to PSA.

## Methods

Nineteen consecutive mCRPC patients from the Department of Urology at Münster University Hospital were enrolled into this study. Patient demographics are included in Table 1.

**Table 1:** Cohort demographics (*n*=19)

**Age (years)**	**68 (54-84)**
*Gleason Scores*	
6	0 (0%)
7a	6 (31.6%)
7b	3 (15.8%)
8	1 (5.3%)
9	6 (31.6%)
10	1 (5.3%)
na	2 (10.4%)
*Metastases*	
Lymph node	12 (63.2%)
Bone	16 (84.2%)
*Prior treatments*	
Surgery	14 (73.7%)
ADT	19 (100%)
Radiation	5 (26.3%)
Abiraterone	13 (68.4%)
Enzalutamide	9 (47.4%)
Lu-PSMA	1 (5.3%)

### Treatment

Patients underwent a median of 10 cycles (range: 5–10) of treatment, with each cycle lasting 2 weeks.

### Follow-up

PSA-response evaluation was available for a median of 4.2 months (range: 1.8–6.2 months). Survival follow-up extended to 14 months (range: 3.6–53 months) and was assessed based on cancer-specific mortality (CSM). In a secondary analysis, we also evaluated OS 12-months after treatment initiation.

### Therapy response

PSA response was based on changes in PSA per the Prostate Cancer Working Group 3-criteria [6]. Three groups were identified, responders (ΔPSA: *≥*50% decrease from baseline), partial responders/stable disease (ΔPSA: −49% decrease to + 25% increase) and non-responders (> + 25% increase).

### Blood sampling

Samples for PSA (EDTA tubes) were collected before the start of therapy (within 1 h of first dose) and before every subsequent cycle. PSA levels were measured according to clinical protocol. Additionally, PROSTest samples (whole blood stored in Wren stabilization tubes) were collected before treatment and after the first cycle. Samples were stored and batch processed per protocol.

### PSA measurements

PSA testing was conducted using the standard clinical PSA assay at Münster University (DVIA Centaur XP Immunoassay System, Siemens Healthcare, Erlangen, Germany).

### PROSTest measurements

Blood samples were collected in Wren’s proprietary RNA stabilization buffer tubes per the sample collection protocol. Specimens were deidentified and coded prior to shipment for analysis. Samples were stored at −80 °C until analyzed in batches by Wren Laboratories. The latter comprises a 2-step protocol (RNA isolation/RT-qPCR) [15]. Transcripts (mRNA) were isolated from whole blood (Mini Blood kit, Qiagen, Valencia CA) and real-time qPCR performed on pre-spotted PCR plates (Life Technologies, Carlsbad CA). Target transcript levels were normalized to *ALG9*, *TOX4* and *TPT1* and quantified using ΔΔC_t_ [13, 14]. Final results are expressed as a risk stratification-based on a 0-100 score for PCa [13, 14]. The cut-off is 50% for risk of disease. The calculated intra- and inter-assay variations for Ct values was 0.83 ± 1.2% and 1.2 ± 1.1%, respectively [14].

### Statistical analyses

Descriptive statistics were used for patient demographics. Data are provided as median (range). PRISM9.4.0 for Windows (GraphPad Software, La Jolla, California, USA, www.graphpad.com) and MedCalc Statistical Software version 20.109 (MedCalc Software bvba, Ostend, Belgium; http://www.medcalc.org; 2017), were utilized. Mann-Whitney U-testing (2-tailed) was undertaken to evaluate whether there were differences between responders/non-responders. Statistical significance was defined as *p* < 0.05. For PFS/response analysis, we included partial responders with responders (*n* = 11 in total) and compared these with PSA-progressors (non-responders, *n* = 8). Kaplan-Meier curves (Log-Rank) were generated from results of changes in PSA (over the course of the therapy) and survival (CSM) to develop progression-free survival (PFS) and OS curves.

The utility of early biomarker changes (after 1 cycle of therapy) in PSA levels and the PROSTest was directly compared to these curves. Changes in either biomarker (PSA or PROSTest) were measured versus the baseline level and provided as a percentage:

$$\frac{\left[\mathrm{Biomarker}\;\mathrm{before}\;\mathrm{cycle}\;2\;-\;\mathrm{Biomarker}\;\mathrm{score}\;\mathrm{at}\;\mathrm{baseline}\;\left(\mathrm{before}\;\mathrm{cycle}\;1\right)\right]}{\left[\mathrm{Biomarker}\;\mathrm{score}\;\mathrm{at}\;\mathrm{baseline}\right]}\ast\;100$$

Changes were either an increase (versus baseline) or a decrease. We evaluated how these changes were associated with PFS and OS (both CSM and 12-months after therapy) using AUROC analysis and KM-survival curves. In a secondary analysis, we examined whether changes in PSA after 3 months were associated with PFS and OS (CSM and 12-month). Associations between binary outputs (e.g., increase/decrease in PSA and outcomes e.g., OS) were evaluated using the phi (φ) co-efficient [16].

## Results

**Fig. 1:**
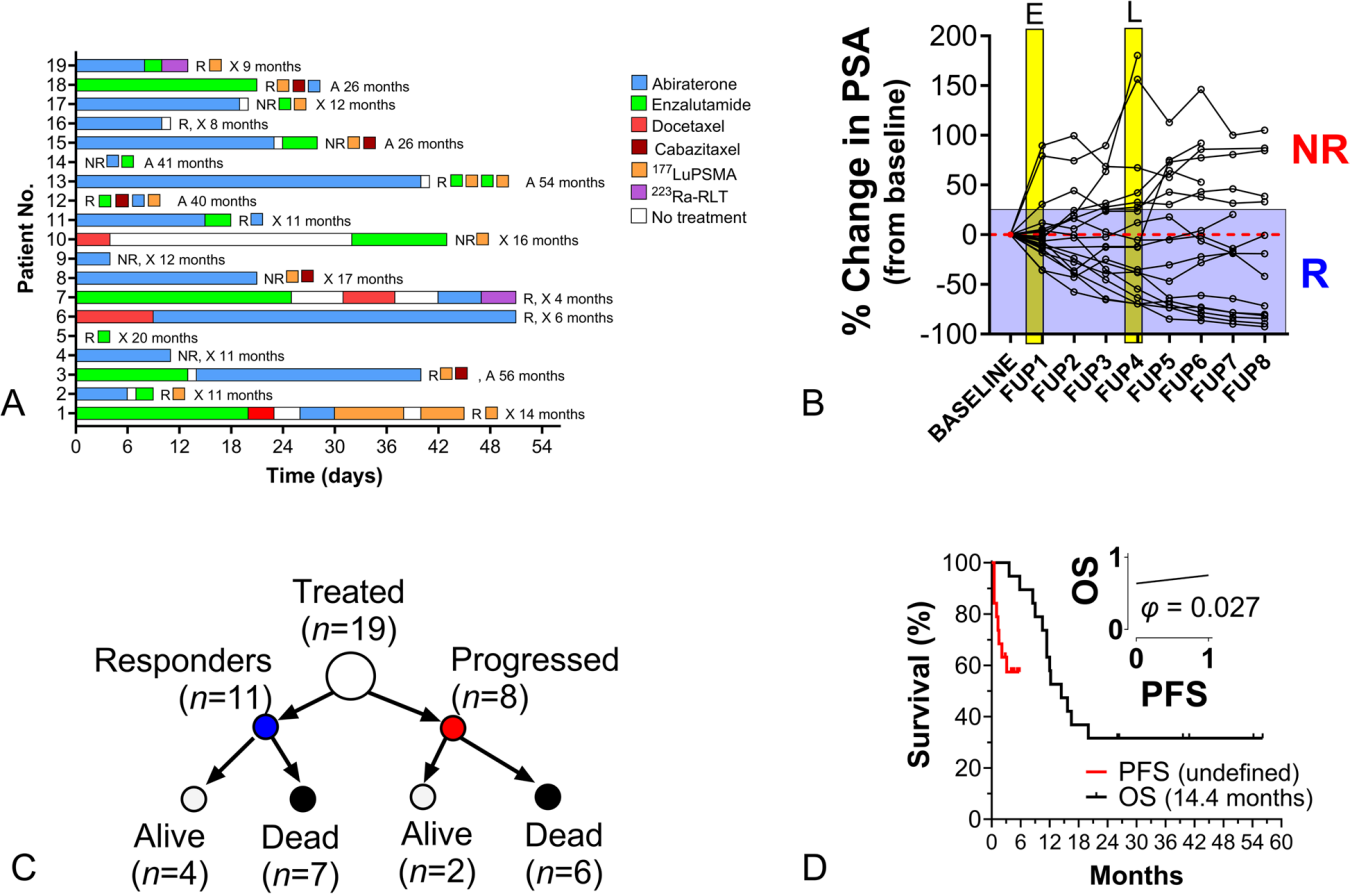
Study cohort and outcomes. **A** Modified Swimmer plots identifying prior therapies. The response to Taxotere (R = responder, N = non-responder), overall survival (A = alive, X = died, at follow-up), subsequent treatments and survival months are included **B**. Changes (%) in PSA from baseline in all 19 patients. E identifies the time point for early PSA change evaluation (after cycle 1) and L identifies the time-point for later evaluation (~ 3 months). *R* = responder (PSA change from baseline of ≤ + 25%); NR = non-responder (PSA change from baseline of > + 25%) **C**. Summary of outcomes based on PSA-PFS and CSM information **D**. PFS (based on PSA changes) and OS survival curves. The phi co-efficient correlation between PFS and OS) is included (*inset*). This was not significant

All patients were mCRPC and were undergoing standard ADT (GNRH analogue injections) at the time of start of treatment. Sixteen (84%) had undergone an average of 2 therapies (range: 1–5, including Abiraterone (*n* = 13) and Enzalutamide (*n* = 8)). Two patients had undergone prior Docetaxal treatment. The average number of treatment months was 22 (range: 4–52 months). Swimmer plots for each subject are included in Fig. 1A. Follow-up treatments and additional treatments as well as OS are included. Median (range) baseline PSA level was 32ng/mL (range: 0.17-2,094) and the median baseline PROSTest score was 86 (range: 73–95) (Table 2).

**Table 2:** Parameters evaluated for response and cancer-specific overall survival

	PFS			OS		
	Responders (*n* = 11)^a^	Non-Responders (*n* = 8)	*p*-value^b,c^	Alive (*n* = 6)	Dead (*n* = 13)	*p*-value^b,c^
Age	68 (58–83)	70 (54–84)	0.95	68 (67–74)	64 (54–84)	0.62
Baseline PSA	40 (6.5–2094)	10 (0.2–1667)	0.098	19 (0.2–58)	40 (8.1–2086)	0.087
Change in PSA (after 1 cycle)	−14% (−38 to + 4.5)	8.6% (−5.5 to + 90)	0.0008	−4.9% (−38 to + 79)	−5.5% (−38 to + 90)	> 0.99
Decrease: Increase (PSA)	9:2	3:5	Chi^2^ = 3.7, *p* = 0.054	3:3	9:4	Chi^2^ = 0.6, *p* = 0.43
Baseline PROSTest	86 (79–95)	83 (73–95)	0.35	86 (82–92)	86 (73–95)	0.46
Change in PROSTest (after 1 cycle)	−6.5% (−60 to −1)	+ 3.5% (−7.7 to + 26)	0.0091	−9.6% (−60 to + 5.9)	−1.5% (−7.2 to + 26)	0.046
Decrease: Increase (PROSTest)	11:0	2:6	Chi^2^ = 11.4, *p* = 0.0007	5:1	8:5	Chi^2^ = 0.85, *p* = 0.35

^a^Includes 3 partial responders; ^b^Mann-Whitney U-test (2-tailed) for age, baseline PSA and change in biomarkers ^c^Chi^2^ (2-tailed) for Decrease: Increase correlation with outcomes. Data is presented as: Median (range)

### Response and overall survival

A total of 8 patients were considered responders to therapy (change in PSA > 50% decrease), 3 had a partial response (PSA: −49 to + 25%) and 8 progressed (PSA > + 25%) (Fig. 1B). The median PFS for the entire group was not reached (Fig. 1C). There were 13 (68%) deaths, with a median OS of 14.4 months (95%CI: 10.5–20). At 12 months, there were 9 deaths. A total of 6 patients progressed (6/8) and died during follow-up as well as 3/3 of the partial responders and 4/8 of the responders. No significant was observed between OS and PFS (*r* = 0.12, *p* = 0.62, Fig. 1D, *inset*). Additionally, age, baseline PSA and baseline PROSTest did not show a meaningful association with either PFS or OS.

### Early biomarker changes and outcomes

PSA and PROSTest levels were assessed at baseline, and changes over the first cycle were analyzed for their relationship to PFS, OS and 12-month OS. Overall, PSA decreased in 12 (63%), while PROSTest decreased in 13 (68%). Concordant changes were noted in 12 patients (increases in 3 (16%) and decreases in 9 (47%)). However, the phi co-efficient identified no association (φ = 0.04).

**Fig. 2:**
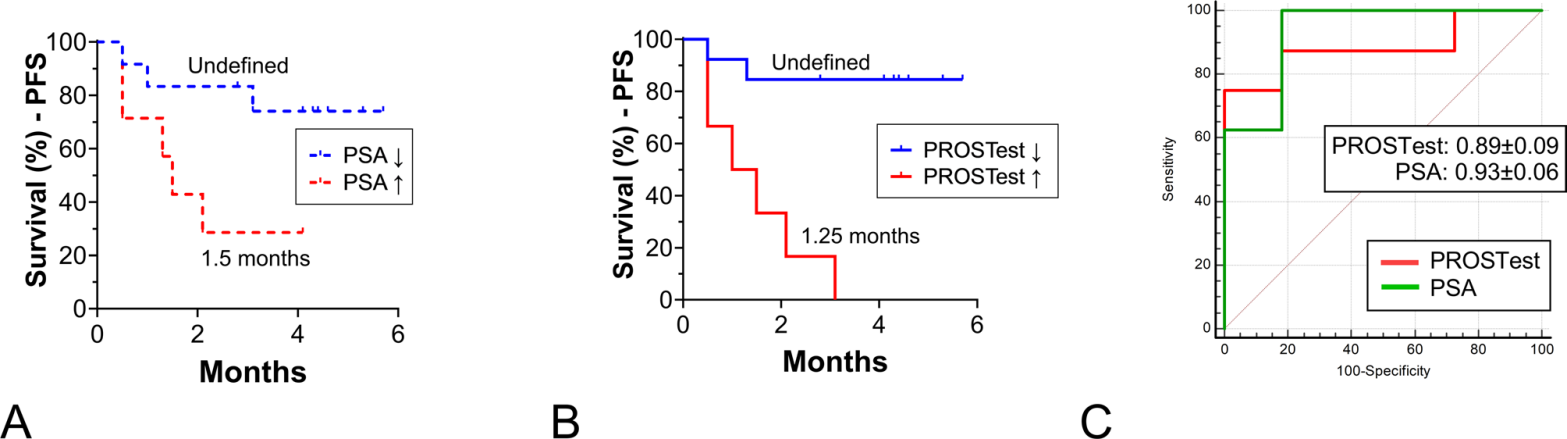
Relationship between biomarker changes (after 1 cycle of treatment) and PFS. **A** Changes in PSA (after 1 cycle of treatment) were associated with median PFS **B**. Changes in PROSTest after 1 cycle of treatment were significantly associated with outcome **C**. AUROC curves for the relationship between the changes (after 1 cycle vs. baseline) in the PROSTest and PSA and PFS

As expected, PSA changes after 1 cycle of treatment, significantly correlated with PSA response (Table 2). Similarly, PROSTest changes also demonstrated a strong correlation with outcomes. Kaplan-Meier survival estimates confirmed both biomarkers were associated with PFS (Fig. 2A-B), though the HR for PROSTest (HR: 0.04, *p* = 0.0003) was more significant than for PSA (HR: 0.2, *p* = 0.04). The AUROC analysis identified no difference between early changes in PSA (0.93 ± 0.25) compared to PROSTest (0.89 ± 0.41) (z-statistic: 0.37, *p* = 0.71, Fig. 2C).

**Fig. 3:**
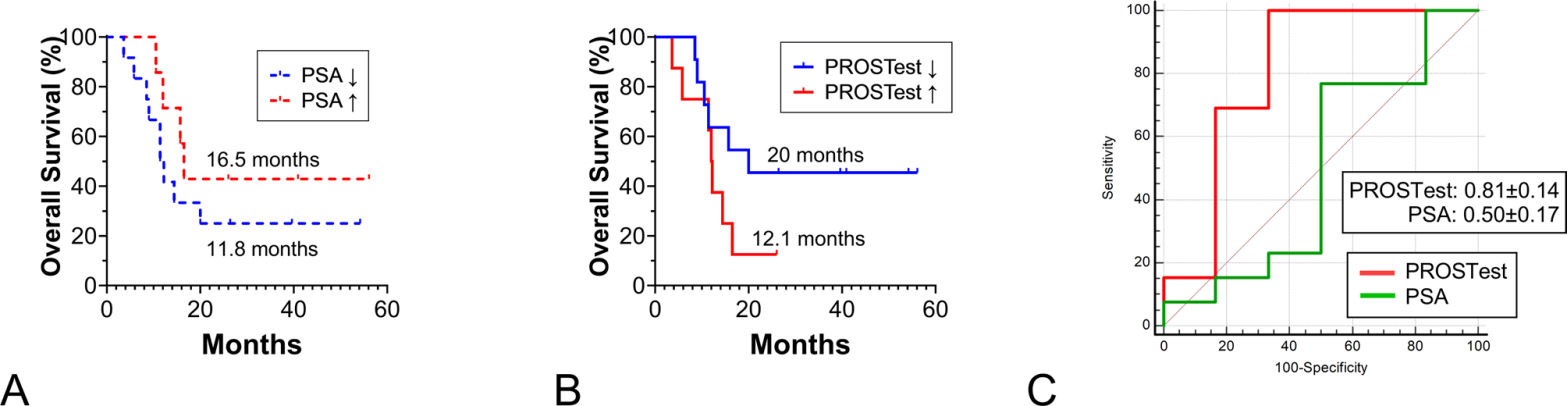
Relationship between biomarker changes (after 1 cycle of treatment) and OS. **A** Changes in PSA (after 1 cycle of treatment) were not (*p* = 0.35) associated with overall survival **B**. Changes in PROSTest after 1 cycle of treatment were significantly (*p* = 0.046) associated with overall survival **C**. AUROC curves for the relationship between the changes (after 1 cycle vs. baseline) in the PROSTest and PSA and OS. Only changes in the PROSTest were significant (*p* = 0.03)

PSA changes after 1 treatment cycle were not associated with OS, PROSTest changes, however, showed a significant correlation with OS (Table 2). Kaplan-Meier survival analysis further confirmed the PROSTest as a valuable response biomarker for OS (Fig. 3A-B). The AUROC analysis identified a significant difference between early changes in PROSTest (0.81 ± 0.14) compared to PSA (0.5 ± 0.17) (z-statistic: 2.13, *p* = 0.03, Fig. 3C).

An evaluation of OS at 12 months identified no relationship between early changes in either PSA (AUC = 0.58 ± 0.14) or the PROSTest (AUC = 0.58 ± 0.14) and demise.

### PSA changes at 3 months and outcomes

Because changes in PSA after 3 months are commonly utilized as a prognostic [17], we also evaluated this parameter and outcome. As expected, there was a significant association between 3-month PSA changes and PFS (AUC = 0.97 ± 0.04, *p* < 0.0001), but there was no relationship with either OS (AUC = 0.67 ± 0.16, *p* = 0.28) or the 12-month OS (AUC = 0.55 ± 0.14, *p* = 0.73) (Table 3). The phi co-efficient identified a strong association between the 3-month PSA and PFS (φ = 0.17) but no association with either OS (φ = 0.027) or the 12-month OS (φ = 0.01). There was a moderate association between the 3-month PSA and changes after the first cycle PSA (φ = 0.101) and with changes in PROSTest after cycle 1 (φ = 0.13).

**Table 3:** Changes in PSA (3-months), PFS, and overall survival

	PSA (Δ3-months)			
	Correlation^a^	AUC^b^	*p*-value	φ co-efficient^c^
PFS	17 (89%)	0.97 ± 0.04	< 0.0001	0.17
12-month OS	9 (47%)	0.55 ± 0.14	0.73	0.01
Overall survival^d^	10 (53%)	0.67 ± 0.16	0.28	0.027

^a^Number correlated (decrease = responder/increase = non-responder or decrease = alive/increase = died) with outcomes

^b^Data is presented as: Median (range).

^c^Parameters are: >0.25 = very strong; >0.15 = strong; >0.10 = moderate; >0.05 = weak; >0 = no or very weak

^d^Overall survival (cancer-specific mortality)

## Discussion

Several genomic and molecular biomarkers have emerged as potential tools for predicting treatment response in PCa, supplementing or even surpassing PSA-based assessments [18]. Circulating tumor cells (CTCs) represent a promising biomarker, as their presence and molecular characterization can provide insights into tumor biology and treatment resistance. Notably, AR-V7 expression in CTCs, detected through Epic Sciences AR-V7 test (commercially available through Johns Hopkins’ Molecular Diagnostics Lab), has been shown to predict resistance to ARPIs, making it a potential tool for identifying patients who may benefit from alternative treatments, such as taxane-based chemotherapy [19]. Tissue-based genomic assays such as Oncotype DX^®^ and Prolaris^®^ have also been integrated into clinical decision-making. Oncotype DX Genomic Prostate Score, a 17-gene panel commercialized by Exact Sciences (formerly Genomic Health, Inc.; Madison WI) [20], aids in stratifying patients with low- and intermediate-risk prostate cancer by assessing tumor aggressiveness, potentially identifying those who may benefit from active surveillance versus immediate intervention. Similarly, Prolaris^®^ (Myriad Genetics, Salt Lake City, UT), a cell cycle progression gene signature test, provides prognostic information on disease progression, helping to refine risk stratification and treatment plans [21].

While these tissue-based evaluations have shown utility in prognosis, their predictive value in determining therapy response remains under investigation. The integration of such biomarkers with liquid biopsy approaches such as PROSTest may enhance the precision of treatment selection, particularly mCRPC, where genomic heterogeneity plays a critical role in therapeutic resistance. In this context, we sought to explore the clinical utility of PROSTest in our cohort, assessing its potential to predict treatment response alongside PSA.

In our cohort, docetaxel was associated with a PSA response in 8 of 19 patients (42%), consistent with prior studies reporting 45–50% efficacy [22, 23]. The overall OS rate was 32% while the 12-month OS was 53%, aligning with previous studies and confirming that our cohort, while small, is clinically representative [24]. We evaluated whether baseline and/or initial changes (after 1 treatment cycle) in PSA and PROSTest could help distinguish responders from non-responders. We also examined their association with cancer-specific (overall) OS.

Baseline PSA and PROSTest levels were not predictive of PSA response in this cohort, which aligns with existing literature recognizing PSA levels as a poor outcome predictor [1, 6]. However, changes in both biomarkers after 1 cycle of docetaxel chemotherapy correlated with treatment response (per guideline-based changes in PSA) [6]. For PSA, the HR was 0.2 (95%CI: 0.04–0.95). For PROSTest, the HR was 0.04 (95%CI: 0.008–0.23). These findings suggest that early biomarker changes, even after a single 1 cycle of therapy, may be useful for predicting response. Interesting, the lower HR for the PROSTest suggests that molecular biomarker assessment may be more accurate than PSA changes in predicting docetaxel outcomes. The AUROC curves were similar, identifying that this finding requires validation in a larger cohort.

Neither baseline PSA levels nor early PSA changes were associated with cancer-specific OS. This likely reflects the PSA “flares” that occur in ~ 30% of patients treated with docetaxel or cabazitaxel chemotherapy [25]. These likely mitigate the value of assessing PSA at early time-points for therapy evaluation. Prior studies have suggested that PSA decline over 3 months may indicate better long-term survival [17]; accordingly, we therefore also examined the value of % PSA change at ~ 3months (versus baseline). In our cohort, while 3-month PSA changes were expectedly associated with PFS [6, 26], there was no relationship with either overall OS or 12-month OS. A secondary analysis (phi coefficient– a statistical measure of the strength of association between dichotomous variables), while affirming the strong PFS association, confirmed no prognostic (survival) association. In contrast, changes after 1 cycle in PROSTest were strongly prognostic, suggesting that gene expression measurements are more accurate than PSA for predicting survival.

Among patients with progressive disease, defined as a PSA increase of > 25% from baseline, 75% died of disease during follow-up. However, 50%of those classified as responders, who experienced a PSA decrease of > 50%, also died. Statistical analysis using Fisher’s exact test (*p* = 0.36) indicated that this difference was not significant, suggesting that early PSA changes alone may not be a reliable predictor of survival outcomes in this cohort.

While baseline PROSTests were not linked to OS, early PROSTest changes were, despite the small sample size. This may reflect that the PROSTest is detecting changes in CTCs, a prognostic for PCa [27], but this is unlikely given the short time-frame between the baseline collection and second sample collection (2 weeks). The PROSTest is measured from a whole blood sample identifying the assay detects circulating tumor RNA from multiple sources (CTCs, cfRNA, exosomes, tumor educated platelets [13]. These results imply that blood-based gene expression marker assays may provide a more holistic approach for tumor biology detection and be more sensitive than ELISA-based assays for detecting tumor response and predicting survival. These data also suggest that the PROSTest may have prognostic value in this setting.

Of note, the concordance in early marker changes was 63% (12/19, 3 increased and 9 decreased). All 3 that increased experienced a short PFS (0.5–2.1 months). All 9 that shared a decrease in biomarkers were responders with a PFS > 4 months. In the 3 that showed an increase PROSTest/decrease PSA after 1 cycle were ultimately progressive and perished within 11–14 months after treatment initiation. Two of 4 with a decreased PROSTest/increase in PSA exhibited OS > 40 months; 2 of the 4 were also responders to the therapy.

Although this study provides preliminary evidence supporting the potential utility of PROSTest as a predictive and prognostic biomarker in mCRPC, several limitations should be acknowledged. First, the small sample size (*n* = 19) limits the generalizability of our findings and increases the potential for statistical variability. Larger, multicenter studies are required to validate these results and establish the reproducibility of PROSTest as a clinical tool. Second, the retrospective nature of this study introduces inherent biases, and prospective trials would be necessary to further assess the predictive value of early PROSTest changes in guiding treatment decisions. Additionally, while PROSTest demonstrated stronger correlations with progression-free and overall survival than PSA, the lack of direct comparisons with other emerging biomarkers, such as circulating tumor cells (CTCs) or AR-V7 status, prevents definitive conclusions regarding its relative performance. Finally, the study primarily focused on docetaxel-treated patients, and it remains unclear whether PROSTest could serve as a predictive marker across other treatment modalities, such as androgen receptor pathway inhibitors or radioligand therapies. Future research should explore PROSTest in a broader clinical context and assess its integration into multimodal biomarker-driven decision-making.

## Conclusions

This pilot study suggests that PROSTest may provide superior predictive and prognostic value compared to PSA in patients with metastatic castration-resistant prostate cancer (mCRPC) undergoing docetaxel therapy. The findings indicate that early changes in PROSTest levels correlate more strongly with treatment response and survival outcomes than PSA, highlighting its potential as a more reliable biomarker in this setting. Further research should focus on, in addition to larger, multicenter studies, assessing its utility across diverse patient populations and treatment regimens. Rigorous validation and standardization will be necessary before integrating PROSTest into routine clinical practice as a biomarker for guiding therapeutic decisions in mCRPC.

## Data Availability

The datasets generated and/or analyzed during the current study are not publicly available due to PHI restrictions but are available from the corresponding author on reasonable request.

## Abbreviations

AAA: Advanced Accelerator Applications
ARPI: Androgen receptor pathway inhibitors
CSM: Cancer-specific mortality
CTC: Circulating tumor cell
HR: Hazard ratio
mCRPC: Metastatic castration-resistant prostate cancer
OS: Overall survival
PCa: Prostate cancer
PFS: Progression-free survival
PSA: Prostate-specific antigen

## References

[1] Gillessen S, Turco F, Davis ID, Efstathiou JA, Fizazi K, James ND, Shore N, Small E, Smith M, Sweeney CJ et al. Management of Patients with Advanced Prostate Cancer. Report from the 2024 Advanced Prostate Cancer Consensus Conference (APCCC). Eur Urol. 2025;87(2):157–216. 10.1016/j.eururo.2024.09.017 39394013

[2] Sartor O, de Bono J, Chi KN, Fizazi K, Herrmann K, Rahbar K, Tagawa ST, Nordquist LT, Vaishampayan N, El-Haddad G, et al. Lutetium-177-PSMA-617 for metastatic castration-resistant prostate cancer. N Engl J Med. 2021;385(12):1091–103. 34161051 10.1056/NEJMoa2107322 PMC8446332

[3] Maurice Dror C, Chi KN, Khalaf DJ. Finding the optimal treatment sequence in metastatic castration-resistant prostate cancer-a narrative review. Translational Androl Urol. 2021;10(10):3931–45. 10.21037/tau-20-1341 PMC8575566 34804836

[4] Azad AA, Kostos L, Agarwal N, Attard G, Davis ID, Dorff T, Gillessen S, Parker C, Smith MR, Sweeney CJ, et al. Combination therapies in locally advanced and metastatic hormone-sensitive prostate cancer. Eur Urol. 2025;87(4):455–67. 39947976 10.1016/j.eururo.2025.01.010

[5] Berthold DR, Pond GR, Soban F, de Wit R, Eisenberger M, Tannock IF. Docetaxel plus prednisone or mitoxantrone plus prednisone for advanced prostate cancer: updated survival in the TAX 327 study. J Clin Oncol. 2008;26(2):242–5. 18182665 10.1200/JCO.2007.12.4008

[6] Scher HI, Morris MJ, Stadler WM, Higano C, Basch E, Fizazi K, Antonarakis ES, Beer TM, Carducci MA, Chi KN, et al. Trial design and objectives for castration-resistant prostate cancer: updated recommendations from the prostate Cancer clinical trials working group 3. J Clin Oncol. 2016;34(12):1402–18. 26903579 10.1200/JCO.2015.64.2702 PMC4872347

[7] Thuret R, Massard C, Gross-Goupil M, Escudier B, Di Palma M, Bossi A, de Crevoisier R, Chauchereau A, Fizazi K. The postchemotherapy PSA surge syndrome. Ann Oncol. 2008;19(7):1308–11. 18356135 10.1093/annonc/mdn062

[8] Conteduca V, Caffo O, Lolli C, Aieta M, Scarpi E, Bianchi E, Maines F, Schepisi G, Salvi S, Massari F, et al. Long-term clinical impact of PSA surge in castration-resistant prostate cancer patients treated with abiraterone. Prostate. 2017;77(9):1012–9. 28429372 10.1002/pros.23357

[9] Song G, Lee C, You D, Jeong IG, Hong JH, Ahn H, Kim CS. Prostate-specific antigen response rate of sequential chemotherapy in castration-resistant prostate cancer: the results of real life practice. Prostate Int. 2013;1(3):125–32. 24223414 10.12954/PI.13024 PMC3814118

[10] Schallier D, Decoster L, Braeckman J, Fontaine C, Degrève J. Docetaxel in the treatment of metastatic castration-resistant prostate cancer (mCRPC): an observational study in a single institution. Anticancer Res. 2012;32(2):633–41. 22287756

[11] Delanoy N, Hardy-Bessard AC, Efstathiou E, Le Moulec S, Basso U, Birtle A, Thomson A, Krainer M, Guillot A, De Giorgi U, et al. Sequencing of taxanes and new androgen-targeted therapies in metastatic castration-resistant prostate cancer: results of the international multicentre retrospective CATS database. Eur Urol Oncol. 2018;1(6):467–75. 31158090 10.1016/j.euo.2018.05.009

[12] Lapini A, Caffo O, Pappagallo G, Iacovelli R, D’Angelillo RM, Vavassori V, Ceccarelli R, Bracarda S, Jereczek-Fossa BA, Da Pozzo L, et al. Monitoring patients with metastatic hormone-sensitive and metastatic castration-resistant prostate cancer: a multidisciplinary consensus document. Cancers. 2019. 10.3390/cancers11121908. 31805687 10.3390/cancers11121908 PMC6966424

[13] Modlin IM, Kidd M, Drozdov IA, Boegemann M, Bodei L, Kunikowska J, Malczewska A, Bernemann C, Koduru SV, Rahbar K. Development of a multigenomic liquid biopsy (PROSTest) for prostate cancer in whole blood. Prostate. 2024;84(9):850–65. 38571290 10.1002/pros.24704

[14] Rahbar K, Kidd M, Prasad V, David Rosin R, Drozdov I, Halim A. Clinical sensitivity and specificity of the prostest in an American cohort. Prostate 2025;85(6):558–66. 10.1002/pros.24858 PMC11934832 39838708

[15] Kidd M, Drozdov IA, Matar S, Gurunlian N, Ferranti NJ, Malczewska A, Bennett P, Bodei L, Modlin IM. Utility of a ready-to-use PCR system for neuroendocrine tumor diagnosis. PLoS One. 2019;14(6): e0218592. 31247038 10.1371/journal.pone.0218592 PMC6597157

[16] Field A. Discovering statistics using IBM SPSS statistics. 5th ed. London: SAGE Publications LTD; 2017.

[17] Ahmed ME, Lee MS, Mahmoud AM, Joshi VB, Gopalakrishna A, Bole R, Haloi R, Kendi AT, Bold MS, Bryce AH, et al. Early PSA decline after starting second-generation hormone therapy in the post-docetaxel setting predicts cancer-specific survival in metastatic castrate-resistant prostate cancer. Prostate Cancer Prostatic Dis. 2024;27(2):334–8. 37935879 10.1038/s41391-023-00751-6

[18] Cucchiara V, Cooperberg MR, Dall’Era M, Lin DW, Montorsi F, Schalken JA, Evans CP. Genomic markers in prostate Cancer decision making. Eur Urol. 2018;73(4):572–82. 29129398 10.1016/j.eururo.2017.10.036

[19] Antonarakis ES, Lu C, Luber B, Wang H, Chen Y, Nakazawa M, Nadal R, Paller CJ, Denmeade SR, Carducci MA, et al. Androgen receptor splice variant 7 and efficacy of taxane chemotherapy in patients with metastatic castration-resistant prostate cancer. JAMA Oncol. 2015;1(5):582–91. 26181238 10.1001/jamaoncol.2015.1341 PMC4537351

[20] Albala D, Kemeter MJ, Febbo PG, Lu R, John V, Stoy D, Denes B, McCall M, Shindel AW, Dubeck F. Health economic impact and prospective clinical utility of oncotype DX^®^ genomic prostate score. Rev Urol. 2016;18(3):123–32. 27833462 10.3909/riu0725 PMC5102928

[21] Cooperberg MR, Simko JP, Cowan JE, Reid JE, Djalilvand A, Bhatnagar S, Gutin A, Lanchbury JS, Swanson GP, Stone S, et al. Validation of a cell-cycle progression gene panel to improve risk stratification in a contemporary prostatectomy cohort. J Clin Oncol. 2013;31(11):1428–34. 23460710 10.1200/JCO.2012.46.4396

[22] Vaishampayan UN, Keessen M, Dreicer R, Heath EI, Buchler T, Árkosy PF, Csöszi T, Wiechno P, Kopyltsov E, Orlov SV, et al. A global phase II randomized trial comparing oral taxane ModraDoc006/r to intravenous docetaxel in metastatic castration resistant prostate cancer. Eur J Cancer. 2024;202: 114007. 38518534 10.1016/j.ejca.2024.114007

[23] Josefsson A, Jellvert Å, Holmberg E, Brasso K, Meidahl Petersen P, Aaltomaa S, Luukkaa M, Verhagen P, de Wit R, Ahlgren G, et al. Effect of docetaxel added to bicalutamide in hormone-naïve non-metastatic prostate cancer with rising PSA, a randomized clinical trial (SPCG-14). Acta Oncol. 2023;62(4):372–80. 37073813 10.1080/0284186X.2023.2199940

[24] Sentana-Lledo D, Chu X, Jarrard DF, Carducci MA, DiPaola RS, Wagner LI, Cella D, Sweeney CJ, Morgans AK. Patient-reported quality of life and survival outcomes in prostate cancer: analysis of the ECOG-ACRIN E3805 chemohormonal androgen ablation randomized trial (CHAARTED). Eur Urol Oncol. 2025;8(1):29–37. 38688766 10.1016/j.euo.2024.04.010 PMC11518880

[25] Sidhu A, Khan N, Phillips C, Briones J, Kapoor A, Zalewski P, Fleshner NE, Chow E, Emmenegger U. Prevalence and prognostic implications of PSA flares during Radium-223 treatment among men with metastatic castration resistant prostate cancer. J Clin Med. 2023. 10.3390/jcm12175604. 37685670 10.3390/jcm12175604 PMC10488545

[26] España S, Ochoa de Olza M, Sala N, Piulats JM, Ferrandiz U, Etxaniz O, Heras L, Buisan O, Pardo JC, Suarez JF, et al. PSA kinetics as prognostic markers of overall survival in patients with metastatic Castration-Resistant prostate Cancer treated with abiraterone acetate. Cancer Manage Res. 2020;12:10251–60. 10.2147/CMAR.S270392 PMC7584507 33116879

[27] Gupta S, Halabi S, Yang Q, Roy A, Tubbs A, Gore Y, George DJ, Nanus DM, Antonarakis ES, Danila DC, et al. PSMA-positive circulating tumor cell detection and outcomes with abiraterone or enzalutamide treatment in men with metastatic castrate-resistant prostate cancer. Clin Cancer Res. 2023;29(10):1929–37. 36897758 10.1158/1078-0432.CCR-22-3233 PMC10192124

